# Modulation of Intestinal Epithelial Glycocalyx Development by Human Milk Oligosaccharides and Non‐Digestible Carbohydrates

**DOI:** 10.1002/mnfr.201900303

**Published:** 2019-06-11

**Authors:** Chunli Kong, Marlies Elderman, Lianghui Cheng, Bart J. de Haan, Arjen Nauta, Paul de Vos

**Affiliations:** ^1^ Immunoendocrinology Group Division of Medical Biology Department of Pathology and Medical Biology University Medical Center Groningen, University of Groningen Hanzeplein 1 9700 RB Groningen The Netherlands; ^2^ FrieslandCampina Stationsplein 4 3818 LE Amersfoort The Netherlands

**Keywords:** glycocalyx, gut barrier function, human milk oligosaccharides, intestinal epithelium, non‐digestible carbohydrates

## Abstract

**Scope:**

The epithelial glycocalyx development is of great importance for microbial colonization. Human milk oligosaccharides (hMOs) and non‐digestible carbohydrates (NDCs) may modulate glycocalyx development.

**Methods and results:**

The effects of hMOs and NDCs on human gut epithelial cells (Caco2) are investigated by quantifying thickness and area coverage of adsorbed albumin, heparan sulfate (HS), and hyaluronic acid (HA) in the glycocalyx. Effects of hMOs (2′‐FL and 3‐FL) and NDCs [inulins with degrees of polymerization (DP) (DP3‐DP10, DP10‐DP60, DP30‐DP60) and pectins with degrees of methylation (DM) (DM7, DM55, DM69)] are tested using immunofluorescence staining at 1 and 5 days stimulation. HMOs show a significant enhancing effect on glycocalyx development but effects are structure‐dependent. 3‐FL induces a stronger albumin adsorption and increases HS and HA stronger than 2′‐FL. The DP3‐DP10, DP30‐60 inulins also increase glycocalyx development in a structure‐dependent manner as DP3‐DP10 selectively increases HS, while DP30‐DP60 specifically increases HA. Pectins have less effects, and only increase albumin adsorption.

**Conclusion:**

Here, it is shown that 2′‐FL and 3‐FL and inulins stimulate glycocalyx development in a structure‐dependent fashion. This may contribute to formulation of effective hMO and NDC formulations in infant formulas to support microbial colonization and gut barrier function.

## 1 Introduction

Breastfeeding is considered the golden standard for supply of nutrients and bioactive molecules to neonates. Important mother milk components for neonatal gastrointestinal development are the human milk oligosaccharides (hMOs). hMOs have been shown to shape the microbiome within the gastrointestinal tract;^1^, ^2^ reduce pathogenic infections by serving as decoy for pathogens;^3^, ^4^ stimulate the immune system of infants, and enhance intestinal barrier function.^5^ However, there are still about 70% of the infants that cannot be solely fed with breastfeeding for a variety of reasons.^6^ These infants receive cow milk derived infant formulas, in which often non‐digestible carbohydrates (NDCs) such as inulins and pectins are included to mimic some functions of hMOs.^7^ To even more closely mimic the compositions of mother milk, significant attempts have been made to synthesize hMOs for supplementation in infant formulas. Some of these molecules can be produced in a cost‐effective way, making application in infant formulas a realistic option.^8^

One important function of hMOs and NDCs in infant formula is stimulation of colonization of the gastrointestinal tract by microbiota and promote development of gut barrier function.^9^, ^10^ A possible way by which this is accomplished is by stimulating the development of the epithelial glycocalyx on gut epithelial cells. The glycocalyx on neonatal gut epithelium provides binding sites for the commensal microorganisms and a well‐developed glycocalyx may prevent adhesion of pathogens and serve as a barrier for luminal toxins and enzymes.^11^, ^12^ The glycocalyx is composed of glycans and proteins. Proteoglycans are generally considered the most important component of the glycocalyx and form the skeleton of the glycocalyx.^13^ In addition, the glycocalyx contains glycosaminoglycan chains which are linked to the core protein of the proteoglycans.^13^ Heparan sulfate (HS) and hyaluronic acid (HA) are the predominant glycosaminoglycan components in glycocalyx.^14^ HS is a linear highly sulfated polysaccharide that is composed of repeating disaccharide units that consist of an O‐sulfated derivative of glucosamine and an uronic acid.^15^ HA is a non‐sulfated disaccharide polymer, lacking the complex chemical structure of HS and is much longer than HS.^16^

If the glycocalyx components are not properly developed it may lead to enhanced chances for gastrointestinal disorders.^17^ The different glycocalyx molecules can contribute in different ways to gut homeostasis. HS can mediate cell signaling by binding numerous extracellular ligands such as fibroblast growth factor and contributes to homeostasis of innate immunity.^18^, ^19^ HA is highly viscous and forms a physical barrier on top of the gut epithelium.^20^, ^21^ In dextran sodium sulfate induced colitis, HA expression is enhanced to support colonic epithelial repair.^22^ Inflammatory bowel disease can be caused by modification of the glycosaminoglycan composition and distribution on gut epithelium.^17^ Proteins adsorbed on glycosaminoglycan also play an important role in the stability of the glycocalyx. When protein adsorption is absent, the interactions within the glycocalyx collapse and barrier function is lost.^14^ Especially absence of albumin induces collapse and shedding of the glycocalyx.^23^

As not much information is available about effects of hMOs and NDCs on glycocalyx development, we performed this study to determine whether and to what extent these molecules influence glycocalyx synthesis by human gut epithelial cells. To this end, we stimulated gut epithelial Caco2 cells for 1 and 5 days with different hMOs and NDCs. Effects were tested on adsorbed albumin, HS, and HA and average thickness and average area coverage of the glycocalyx layer components on gut epithelial cells. We tested effects of the hMOs 2′‐FL (2′‐fucosyllactose; Fucα1, 2‐ Galβ1, 4Glc) and 3‐FL (3‐fucosyllactose; Galβ1, 4(Fucα1, 3) Glc). Effects were compared with molecules that are often used as substitutes for hMOs in infant formula. To this end we tested inulins with three different degrees of polymerization (DP) (DP3‐DP10, DP10‐DP60, DP30‐60) and pectins with three different degrees of methylation (DM) (DM7, DM55, DM69) in order to gain insight in chemical structure–effects relationships.

## 2 Experimental Section

### 2.1 Carbohydrates

The human milk oligosaccharides (hMOs), 2′‐FL was provided by FrieslandCampina Domo (the Netherlands) and 3‐FL was provided by Glycosyn LLC (Woburn, MA, USA). Commercially extracted chicory inulins with different degrees of polymerization range (DP3‐DP10, DP10‐DP60, and DP30‐60) were provided by Sensus (the Netherlands). DP3‐DP10 is of the highly soluble powdered Frutafit CLR inulins, which was produced from partially hydrolyzed chicory inulin. DP10‐DP60 and DP30‐DP60 are of the moderate soluble powdered Frutafit TEX! inulins. Lemon originated pectins with different degrees of methylation (DM7, DM55, and DM69) were obtained from CP Kelco (Denmark). Endotoxin levels in the eight samples were analyzed by endotoxin detection kit (Thermo Fisher Scientific), and all fell below the endotoxin detection level of 0.1 ng mL^−1^. All of the carbohydrates were dissolved to 2 mg mL^−1^ in cell culture media before stimulation. After optimization by comparing concentrations of 2, 5, and 10 mg mL^−1^ in a pilot study, a slight, gradual increasing effect was observed when the concentration increased. As 2 mg mL^−1^ elicited a response allowing quantification in time, 2 mg mL^−1^ was finally chosen.^24^, ^25^

### 2.2 Cell Culture

Human colon carcinoma Caco2 cells were incubated with 5% CO_2_ at 37 °C in Dulbecco's Modified Eagle Medium (DMEM, Lonza), supplemented with 10% v/v fetal calf serum (FCS, Invitrogen), 1% v/v non‐essential amino acid (NEAA, Sigma), 50 U mL^−1^ Penicillin (Sigma), 50 µg mL^−1^ Streptomycin (Sigma), and 2.5% v/v HEPES (Sigma) to maintain a stable pH environment. The cells density was adjusted to 1.6 × 10^4^ mL^−1^ before being seeded onto the 8‐well Lab‐Tek Chamber Slide (w/Cover, Nunc, Thermo Fisher Scientific). To improve the adhesion of cells to the slide, 200 µL of poly‐l‐lysine solution (Sigma) was applied to pre‐coat the slide. After 2 days of growth, the cells were stimulated with the eight types of carbohydrates for 1 or 5 days.

### 2.3 Immunofluorescence Staining

For staining of albumin, the following procedure was applied. After stimulation with carbohydrates, Caco2 monolayers were washed with 1× Dulbecco's phosphate‐buffered saline (DPBS), fixed with 2% paraformaldehyde/0.1% glutaraldehyde for 30 min, and blocked with 2% donkey serum for 30 min at room temperature. After overnight incubation with anti‐albumin (rabbit IgG, 1:150, Invitrogen) at 4 °C, cells were washed with 1× DPBS for three times and incubated with Alexa Fluor 555 donkey anti‐rabbit secondary antibody (1:400, molecular probes) in the dark for another 30 min, followed by washing three times with 1× DPBS.

The HS staining procedure was the same as the staining for albumin, except that the cells were incubated overnight with Ab‐Heparan Sulfate F58‐10E4 (1:100, amsbio) at 4 °C, and after washing three times with 1 × DPBS, the cells were incubated with Alexa Fluor 488 donkey anti‐mouse secondary antibody (1:100, molecular probes) in the dark for 30 min.

HA staining was also performed in virtually the same way as the staining for albumin, but the cells were blocked with 2% goat serum for 30 min and incubated overnight with hyaluronic acid binding protein (HABP, 50 µg mL^−1^, Calbiochem) at 4 °C. After three times washing with 1 × DPBS, the cells were incubated with Streptavidin FITC (1:100, eBioscience) in the dark for 30 min.

DAPI (1:5000, Sigma) staining in the dark for 10 min was applied to stain the nuclei of Caco2 cells. This was done after the staining of the glycocalyx layer components, followed by washing three times with 1 × DPBS. Then the chamber frame was removed, cells on the slide were mounted with CitiFluor (Electron Microscopy Sciences), and covered with glass coverslip. For all of the three glycocalyx layer components, the control groups were incubated with 1 × DPBS overnight at 4 °C instead of the primary antibodies. For the negative control group, the primary and secondary antibodies were both replaced with 1 × DPBS.

### 2.4 Confocal Microscopy

All the images were captured with a Leica SP8 confocal laser microscope (Leica Microsystems, Wetzlar, Germany) with the 64×/1.4 oil DIC objective. Albumin was excited at 555 nm and emitted at 580–650 nm (red); HS and HA were excited at 488 nm, and emitted at 500–580 nm (green); DAPI was excited at 405 nm, and emitted at 420–460 nm (blue). *Z*‐stack (512‐ × 512‐ pixel resolution × 8 bit) images of each field of view (FOV, 246.51 × 246.51 µm^2^) were taken with a step length of 1.0 µm from the bottom to the top of the monolayer. At least three images were taken of each sample in one experiment.

**Figure 1:**
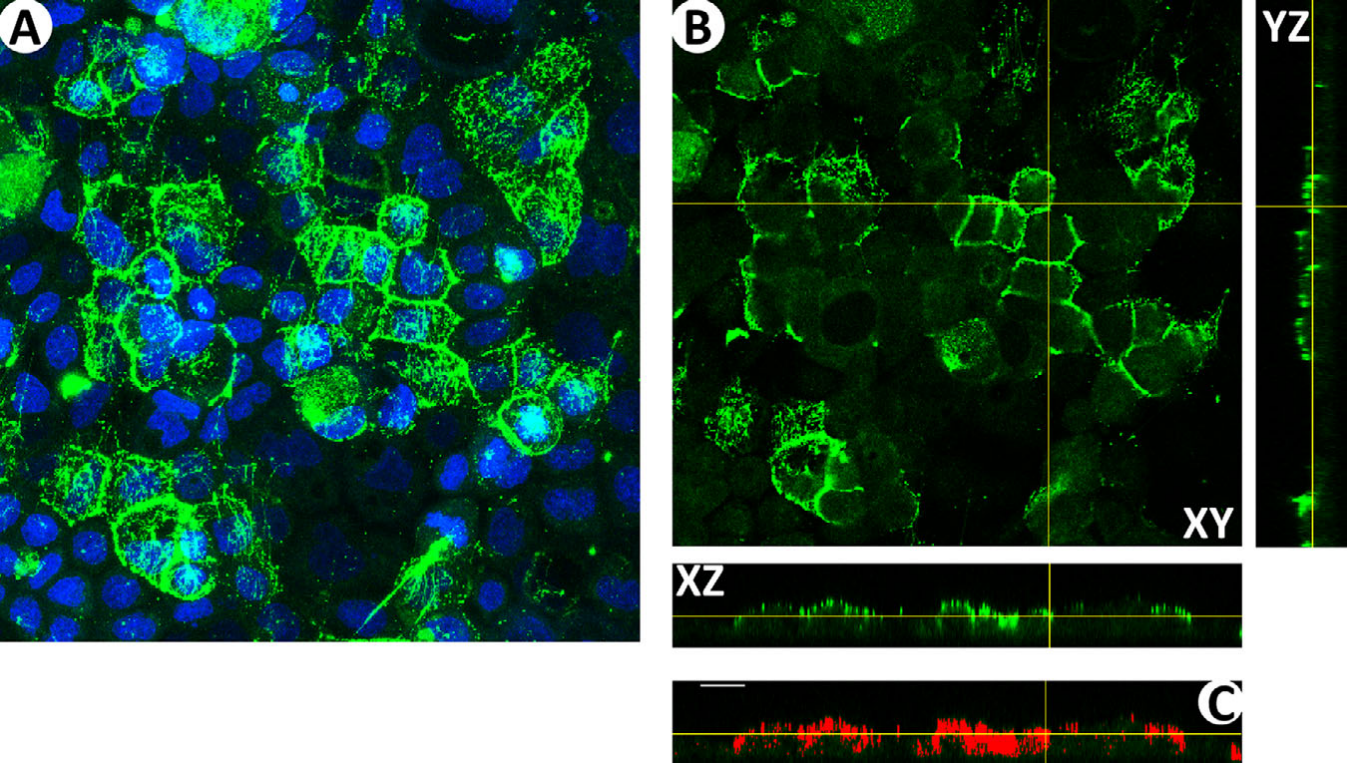
Glycocalyx thickness analysis. A) The merged images of the maximum intensity from the *Z*‐projection of the FITC (green) and DAPI (blue) channels. B) The orthogonal views of the FITC channel. C) We applied the threshold value determined from the corresponding control and negative control groups to include all of the green channel of the *XZ*‐axis of this plane. At least 10 *XZ* and *YZ* slices were chosen for quantification of each image. Scale bar = 20 µm.

The average thickness of the glycocalyx components was quantified according to the method of using the ImageJ software (Version 1.51n; National Institutes of Health, USA) as shown in **Figure** 1.^26^ The staining of HA is shown in Figure 1A as an example of merged images of the maximum intensity plane from the *Z*‐projection of the FITC (green) and DAPI (blue) channels. The green and blue channels were split, and then focus was turned to the orthogonal views of the green channel (Figure 1B). Along the *XZ*‐axis, the threshold value can be adjusted to cover the green channel with red color (Figure 1C). The threshold values for all the images quantification were determined from the corresponding control and negative control groups as follows: the pixel intensity histograms from the maximum intensity *Z*‐projections of the control and negative control were introduced to one curve, and the intersection point was defined as the threshold value that was applied for the data analysis. At least 10 *XZ* and *YZ* slices were chosen of each image to do the quantification. As for one experiment, at least three images were taken, and at least six individual experiments were done. There were at least 360 slices involved in the average thickness quantification. The average thickness of each slice of the green channel was calculated as Equation (1).

(1) Average  thickness = Total  area  Total  length

All of the *Z*‐stack images taken by the Leica SP8 confocal microscope (at least 18 images for each sample) were applied to quantify the average area of each glycocalyx layer component using IMARIS software (Version 8.0, Bitplane, Switzerland). There were two channels in total. The blue (DAPI) channel was for the quantification of the total number of the cells of each image; The green (FITC, for example) channel can be split out to quantify the total area of the staining. The threshold value of the green channel was adjusted to include the whole staining. The average area was expressed as Equation (2).

(2) Average  area = Total  area  Total  number  of  cells

### 2.5 Statistical Analysis

Data analysis was done by GraphPad Prism 6 statistical software (GraphPad Prism Software Inc. San Diego, CA, USA). The Kolmogorov–Smirnov test was applied to determine normality of data distribution. Results were expressed as mean ± SD. All data were finally analyzed with Kruskal–Wallis test of one‐way ANOVA. Significant difference level was defined as *p* < 0.05 (*p < 0.05, **p < 0.01), *p* < 0.1 was considered to be a statistical trend.

## 3 Results

The glycocalyx layer of gut epithelial cells is the primary site for adhesion of commensal bacteria. Its compositions and development are important for building up of the intestine barrier and a normal gut microbiota.^27^ As human milk oligosaccharides are involved in stimulating gut microflora,^28^ we tested whether this may happen via stimulation of the development of the glycocalyx on gut epithelial cells that form anchoring points for gut bacteria.

The glycocalyx major constituents are HS and HA chains.^16^ In the human body albumin provides stability to the glycocalyx. Albumin is synthesized in the liver, and adsorbed on gut epithelial cells.^29^ It can be found in the small intestine^30^ and contributes to the structure of the glycocalyx layer.^31^ Albumin was also present in the culture medium and could therefore also be studied. We tested the effects of the hMOs 2′‐FL and 3‐FL, inulins with DPs of DP3‐DP10, DP10‐DP60, and DP30‐60, and lemon pectins with DMs of DM7, DM55, and DM69. The average thickness and average area of albumin, HS, and HA components around the cells that were covered with glycocalyx were quantified. This was done after 1 day and 5 days of culture to follow the development of the glycocalyx when the gut epithelial cells were exposed to the hMOs and NDCs.

### 3.1 The Glycocalyx Layer Compositions of Caco2 Cells After 1 Day and 5 Days Exposure to hMOs

The hMOs 2′‐FL and 3‐FL were tested first. After 1 day and 5 days stimulation with 2 mg mL^−1^, the cells were stained for albumin, HS, and HA on Caco2 epithelial cells.

**Figure 2:**
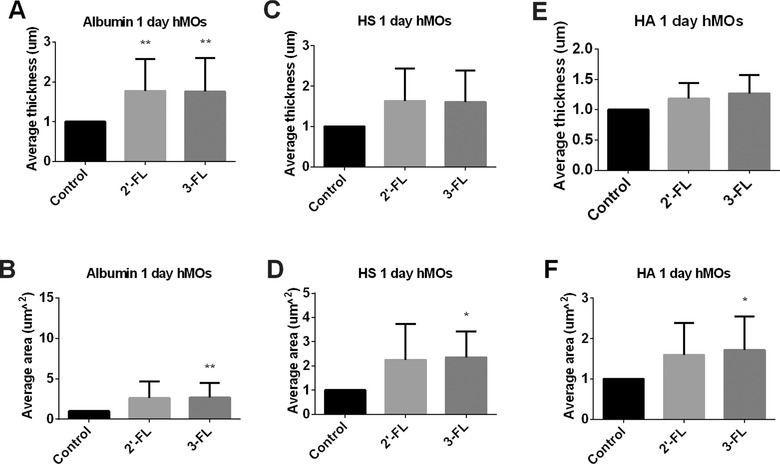
The glycocalyx layer compositions of Caco2 cells were modified by hMOs at 1 day of culture. Caco2 cells were stimulated with hMOs. These were 2'‐FL and 3‐FL tested at 2 mg mL^−1^ for 1 day. Untreated Caco2 cells served as controls. After incubation, we applied immunofluorescence to label the glycocalyx layer components albumin, HS, and HA. 3D images were taken with a Leica SP8 confocal laser microscope. The average thickness of A) albumin, C) HS, and E) HA were quantified using ImageJ software of the 3D images. The average area of cells covered with B) albumin, D) HS, and F) HA were measured with IMARIS software of the 3D images. All data were expressed as mean with standard deviation from six replicates. Statistical significance was tested using one‐way ANOVA (*p < 0.05, **p < 0.01).

After 1 day of stimulation, both 2′‐FL and 3‐FL hMOs significantly increased the thickness of the albumin layer in the glycocalyx (*p* < 0.01, **Figure** 2A). The average area coverage by albumin was almost doubled by the hMOs but only 3‐FL reached statistical significant levels (*p* < 0.01, Figure 2B).

There was a tendency that the average thickness of HS was also increased by the hMOs after 1 day of stimulation (*p* < 0.10, Figure 2C). The average area of cells that were covered with HS was increased by the hMOs, but the magnitude of increase was hMOs‐type dependent and only reached statistical significance with 3‐FL (*p* < 0.05, Figure 2D).

There was no effect on the average thickness of HA of the two hMOs (Figure 2E). However, the average area of cells covered with the HA‐component was increased by both 2′‐FL and 3‐FL but only 3‐FL reached statistical significance (*p* < 0.05, Figure 2F).

**Figure 3:**
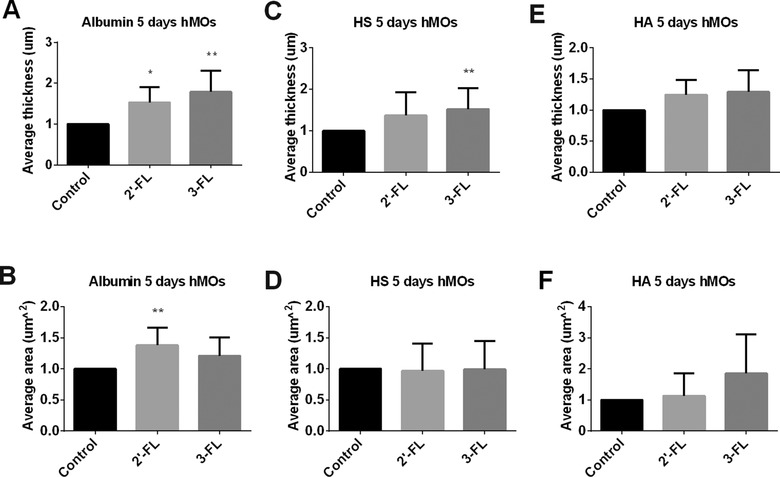
The glycocalyx layer compositions of Caco2 cells were modified after 5 days of exposure to hMOs. Caco2 cells were stimulated with hMOs. These were 2'‐FL and 3‐FL tested at 2 mg mL^−1^ for 5 days. Untreated Caco2 cells served as controls. After incubation, we applied immunofluorescence to label the glycocalyx layer components albumin, HS, and HA. 3D images were taken with a Leica SP8 confocal laser microscope. The average thickness of A) albumin, C) HS, and E) HA were quantified using ImageJ software of the 3D images. The average area of cells covered with B) albumin, D) HS, and F) HA were measured with IMARIS software of the 3D images. All data were expressed as mean with standard deviation from six replicates. Statistical significance was tested using one‐way ANOVA (*p < 0.05, **p < 0.01).

A duration of 5 days exposure to the hMOs resulted in a stable glycocalyx layer. The average thickness of albumin was significantly improved by the 2′‐FL (*p* < 0.05, **Figure** 3A) and 3‐FL (*p* < 0.01, Figure 3A). The average area of cells covered with albumin was also increased by the hMOs, while 2′‐FL reached statistical significance (*p* < 0.01, Figure 3B).

Both 2′‐FL and 3‐FL increased the average thickness of the glycocalyx layer that contains HS, but only 3‐FL hMO showed significant effects (*p* < 0.01, Figure 3C). The average area that was covered with HS component was not influenced by the hMOs (Figure 3D).

The 3‐FL almost doubled the average area of cells that were covered with HA but did not reach statistical significance (Figure 3F).

### 3.2 The Glycocalyx Layer Compositions of Caco2 Cells After 1 Day and 5 Days Exposure to Inulins

Inulins are often used as substitute for hMOs in infant formulas. Inulins vary in composition in different infant formulations and were therefore tested in three different DP compositions. Inulins with DP3‐DP10, DP10‐DP60, and DP30‐60 were incubated with Caco2 cells at a concentration of 2 mg mL^−1^. After 1 day and 5 days of culture, the cells were stained and analyzed for the average thickness and average area of the glycocalyx.

**Figure 4:**
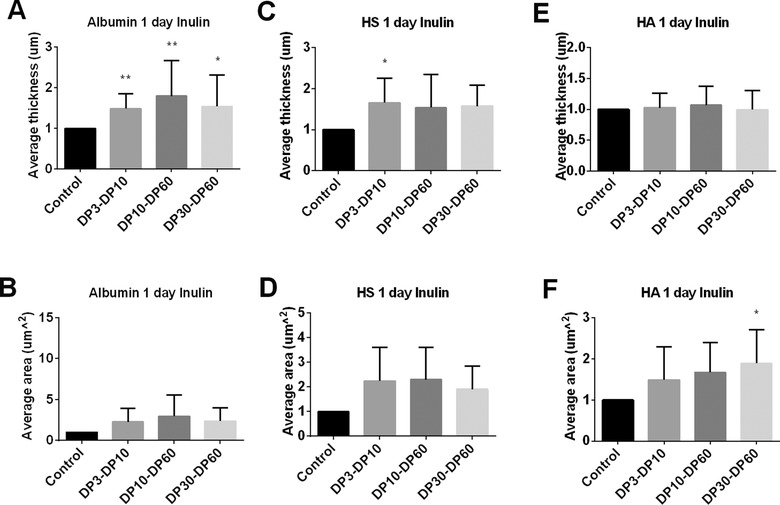
The glycocalyx layer compositions of Caco2 cells were modified by inulins at 1 day of culture. Caco2 cells were stimulated with different chain length of inulins. These were inulins with DP3‐DP10, DP10‐DP60, and DP30‐60 tested at 2 mg mL^−1^ for 1 day. Untreated Caco2 cells served as controls. After incubation, we applied immunofluorescence to label the glycocalyx layer components albumin, HS, and HA. 3D images were taken with a Leica SP8 confocal laser microscope. The average thickness of A) albumin, C) HS, and E) HA were quantified using ImageJ software of the 3D images. The average area of cells covered with B) albumin, D) HS, and F) HA were measured with IMARIS software of the 3D images. All data were expressed as mean with standard deviation from six replicates. Statistical significance was tested using one‐way ANOVA (*p < 0.05, **p < 0.01).

A pronounced development of the glycocalyx was already observed after day 1. After 1 day of stimulation, DP3‐DP10, DP10‐DP60, and DP30‐60 inulins all significantly increased the average thickness of the glycocalyx layer that contains the albumin (*p* < 0.05, **Figure** 4A). There was no DP dependent effect of the inulins on adsorbed albumin.

The average thickness of the HS component was also increased by the inulins but only reached statistical significance with inulins DP3‐DP10 (*p* < 0.05, Figure 4C). The average area that was covered by HS was almost doubled by the inulins but was not more than a statistical trend (*p* < 0.1, Figure 4D).

The average thickness of the HA was not influenced by the inulins (Figure 4E) but the average coverage of the cells with HA was increased in a DP‐dependent manner. The long chain DP30‐DP60 had the most pronounced effect and almost doubled HA on the cells (*p* < 0.05, Figure 4F).

**Figure 5:**
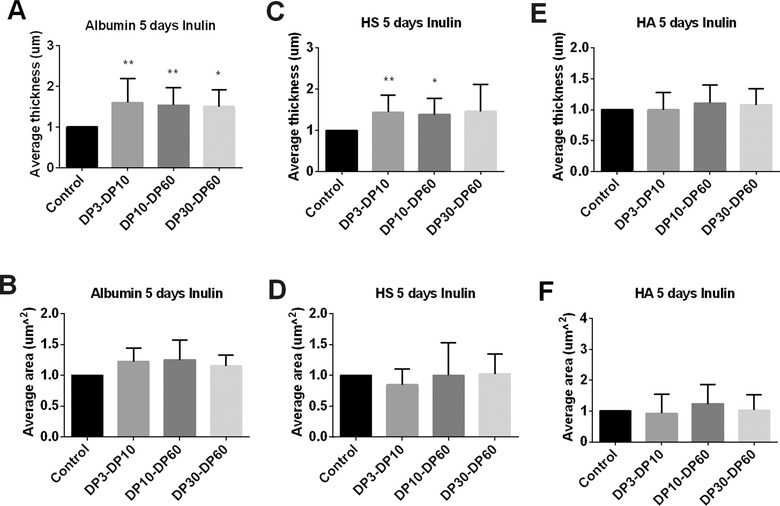
The glycocalyx layer compositions of Caco2 cells were modified after 5 days of exposure to inulins. Caco2 cells were stimulated with different chain length of inulins. These were inulins with DP3‐DP10, DP10‐DP60, and DP30‐60 tested at 2 mg mL^−1^ for 5 days. Untreated Caco2 cells served as controls. After incubation, we applied immunofluorescence to label the glycocalyx layer components albumin, HS, and HA. 3D images were taken with a Leica SP8 confocal laser microscope. The average thickness of A) albumin, C) HS, and E) HA were quantified using ImageJ software of the 3D images. The average area of cells covered with B) albumin, D) HS, and F) HA were measured with IMARIS software of the 3D images. All data were expressed as mean with standard deviation from six replicates. Statistical significance was tested using one‐way ANOVA (*p < 0.05, **p < 0.01).

A period of 5 days exposure resulted in a more pronounced effect of the inulins. DP3‐DP10, DP10‐DP60, and DP30‐DP60 inulins all significantly increased the average thickness of the albumin component (*p* < 0.05, **Figure** 5A), while they only showed a slight increased effect on the average coverage of the albumin (Figure 5B).

The average thickness of HS component was also increased by the inulins after 5 days stimulation, but it only reached statistical significance after DP3‐DP10 (*p* < 0.01, Figure 5C) and DP10‐DP60 (*p* < 0.05, Figure 5C) inulin exposure. The average thickness of HA was not influenced after 5 days exposure to inulin (Figure 5E).

### 3.3 The Glycocalyx Layer Compositions of Caco2 Cells After 1 Day and 5 Days Exposure to Pectins

Pectin‐derived oligosaccharides are another NDC source used as supplement in infant formula.^7^ Since the biological functions are reported to be DM dependent,^32^ pectins with different methylation degrees of DM7, DM55, and DM69 were also applied to stimulate Caco2 cells at a concentration of 2 mg mL^−1^. Cells were harvested and stained for glycocalyx layer components after 1 day and 5 days incubation.

**Figure 6:**
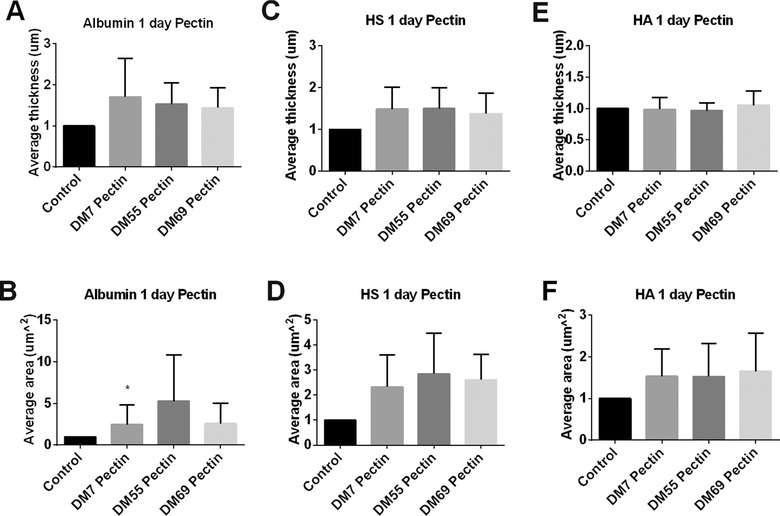
The glycocalyx layer compositions of Caco2 cells were modified by pectins at 1 day of culture. Caco2 cells were stimulated with different methylation degrees of pectins. These were pectins with DM7, DM55, and DM69 tested at 2 mg mL^−1^ for 1 day. Untreated Caco2 cells served as controls. After incubation, we applied immunofluorescence to label the glycocalyx layer components albumin, HS, and HA. 3D images were taken with a Leica SP8 confocal laser microscope. The average thickness of A) albumin, C) HS, and E) HA were quantified using ImageJ software of the 3D images. The average area of cells covered with B) albumin, D) HS, and F) HA were measured with IMARIS software of the 3D images. All data were expressed as mean with standard deviation from six replicates. Statistical significance was tested using one‐way ANOVA (*p < 0.05).

After 1 day stimulation, the average thickness of the albumin was almost doubled, but this did not reach statistical significance (**Figure** 6A). The average area of the cells covered with albumin was increased by pectins in a DM dependent manner, in which the low DM7 pectin significantly increased the average area of albumin in the glycocalyx layer (*p* < 0.05, Figure 6B).

The average thickness of the glycocalyx layer containing HS was also increased but never reached statistical significance (Figure 6C). The pectins didn't influence the average thickness of HA. Although the average area of cells covered with HA was increased by pectins, we didn't observe any significant difference (Figure 6F).

**Figure 7:**
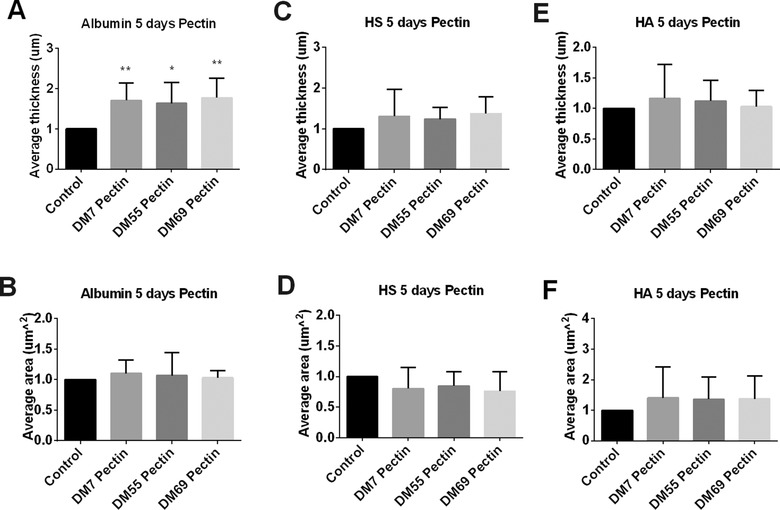
The glycocalyx layer compositions of Caco2 cells were modified after 5 days of exposure to pectins. Caco2 cells were stimulated with different methylation degrees of pectins. These were pectins with DM7, DM55, and DM69 tested at 2 mg mL^−1^ for 5 days. Untreated Caco2 cells served as controls. After incubation, we applied immunofluorescence to label the glycocalyx layer components albumin, HS, and HA. 3D images were taken with a Leica SP8 confocal laser microscope. The average thickness of A) albumin, C) HS, and E) HA were quantified using ImageJ software of the 3D images. The average area of cells covered with B) albumin, D) HS, and F) HA were measured with IMARIS software of the 3D images. All data were expressed as mean with standard deviation from six replicates. Statistical significance was tested using one‐way ANOVA (*p < 0.05, **p < 0.01).

After 5 days stimulation, the pectins all significantly increased the average thickness of albumin (*p* < 0.05, **Figure** 7A). However, the average area coverage of albumin was not influenced by the pectins (Figure 7B). There were no effects on the development of HS and HA components (Figure 7C–F).

## 4 Discussion

The intestinal epithelial glycocalyx is acknowledged for its function as initial adherence site for microbiota in infants and for its initial barrier function toward pathogen invasion and luminal toxins.^11^, ^12^, ^33^ Despite this pivotal function of the epithelial glycocalyx only minor information is available about the gut epithelial glycocalyx composition and possible effects of dietary components on the epithelial glycocalyx. Here we show, to the best of our knowledge for the first time, that hMOs as well as commonly used NDCs in infant formula (inulins and pectins) do impact the average thickness and average area coverage of the glycocalyx on intestinal epithelial cells.^7^, ^8^

hMOs and NDCs have for long been recognized to support host–microbe interactions by serving as prebiotics, decoy for pathogens, or antimicrobial agent.^3^, ^27^, ^34^ However, they may also impact intestinal epithelial cells directly. Here, we studied the quantitative effects and differences in impact of hMOs and NDCs on individual glycocalyx components on the intestinal absorptive enterocytes Caco2 cell‐line.^35^ The reason for choosing this cell‐type and not other commonly used cell‐lines such as the HT29‐MTX cell‐line is that Caco2 is not dedicated to mucus production such as HT29‐MTX that has a clear intestinal epithelial mucus‐secreting goblet‐phenotype.^36^ Caco2 can produce the glycocalyx at a sufficient and appropriate rate to allow quantification of time‐dependent effects of the studied molecules.

The hMOs and NDCs investigated here do stimulate the development of the glycocalyx on intestinal epithelial cells. This may be a possible mechanism for the observed enhancing effects of hMOs and NDCs and its beneficial effects on gut microbiota that need the glycocalyx to adhere to the gut epithelium, with subsequent positive effects on fermentation products short chain fatty acids (SCFAs) and vitamins.^37^ Also, it may be a possible mechanistic explanation for the hMOs and NDCs induced enhanced gut barrier function.^5^, ^10^, ^38^ As shown in our current study the glycocalyx reinforcing effects of the tested food ingredients are due to direct effects on the epithelial cells and not induced by possible microbiota effects as all tests were performed in the absence of bacteria. Effects on the glycocalyx are dependent on the structure of the hMOs and NDCs as discussed below.

We show that the hMOs 2′‐FL, 3‐FL as well as inulins with DP3‐DP10, DP10‐DP60, and DP30‐DP60 significantly increased the average thickness of adsorbed albumin on gut epithelial cells within 1 day of stimulation. These effects in albumin uptake in the glycocalyx are long lasting and still observed after 5 days. Pectins had less effects at day 1. Only after 5 days stimulation with DM7, DM55, and DM69 pectins, a significant effect on albumin adsorption was observed. An increase in albumin adsorption in the glycocalyx is considered to provide more stability of the glycocalyx as other components including HS and HA are better integrated into the glycocalyx structure by the adsorbed protein.^14^, ^26^ Also increased albumin adsorption may contribute to the anti‐pathogenic effects of the glycocalyx as it has been shown that a supplement of albumin results in less adhesion of, for example, *Staphylococcus epidermidis*, *Staphylococcus aureus, Pseudomonas aeruginosa*.^39^, ^40^ An increased albumin adsorption also contributes to enhanced barrier function as it protects during volume resuscitation in hydroxyethyl starch solutions against adverse effects of hydroxyethyl starch on intestinal cells, metabolic functions, fluid shifts, and epithelial barrier permeability.^41^ This suggests that the enhancing effects on albumin adsorption of hMOs and NDCs may not only strengthen the glycocalyx structure but also protect against pathogen adhesion and maintain under luminal changes the gut epithelial barrier.

hMO 3‐FL but not 2′‐FL had a thickness increasing effect on HS after 5 days of stimulation. Of the tested NDCs only the short chain inulin DP3‐DP10 had such an effect on HS and significantly increased the average thickness of HS on both day 1 and 5. Even though only minor information is available on functional effects of HS increase on intestine epithelial cells, it has been shown for endothelial cells that a thicker HS layer provides stronger hydraulic resistance.^42^ HS and HA are the major components of glycosaminoglycan in the glycocalyx.^14^ Both of them can regulate mechanotransduction and maintain gut barrier integrity.^19^, ^43^ Interestingly, just like with HS also HA in the glycocalyx was increased by 3‐FL and not by 2′‐FL after 1 day of stimulation. Of the NDCs tested only the long chain inulin DP30‐60 increased HA on day 1. An increased HA expression is associated with a defense mechanism against intestinal injury and inflammation.^21^, ^22^ In response to endoplasmic reticulum stress, HA accumulates to form cable‐like structure which can serve as binding sites for leukocytes.^44^ Increased HA expression may also contribute to expedited signaling with gut microbiota, as HA binds to toll‐like receptors (TLR) 2 and 4, which are important receptors in regulation of host responses to both commensal and pathogenic bacteria within the gastrointestinal tract.^21^ The above observations support that both hMOs and the tested NDCs have glycocalyx modifying effects, which may be instrumental in providing binding sites for commensals.^34^

Thus, our data demonstrate that hMOs stimulate the glycocalyx development in a structure‐dependent manner. The reason for choosing 2′‐FL and 3‐FL in our studies is that these molecules can be produced in sufficient amounts to allow application of these hMO molecules in infant formula. In addition, 2′‐FL is one of the most abundant hMOs in mother milk.^8^ Our data illustrate that even the structurally related 2′‐FL and 3‐FL had different effects in the glycocalyx. The 2′‐FL hMO impacted the average thickness of the albumin layer at day 1 and 5 while 3‐FL not only increased this average thickness of albumin but also improved the average area coverage of albumin after 1 day. This took more time with 2′‐FL that needed 5 days to significantly increase the average area of albumin. The average area coverage of HS and HA were significantly increased by 3‐FL on day 1, while there is no effect after 5 days stimulation. The 3‐FL also significantly increased the average thickness of HS, but no effects were observed with 2′‐FL. Such a large difference in effect is rather surprising as 2′‐FL and 3‐FL share the same core structure and only differ in that the L‐fucose of 2′‐FL is fucosylated to galactose, while the L‐fucose of 3‐FL is fucosylated to glucose.^8^ The 3′‐sialyllactose (3′‐SL) is another trisaccharide hMO that shares the same core structure with 2′‐FL and 3‐FL, but differ in a sialylated lactose on 3‐FL.^8^ Incubation of 3′‐SL together with Caco2 cells have been reported to reduce sialic acid and lactosamine expression.^25^ Our data suggest that 2′‐FL and 3‐FL are also able to directly modulate the intestinal epithelial cells surfaces, and the difference in fucosylation site has a significant impact on the glycocalyx synthesis machinery in epithelial cells.

The effects of inulins on the epithelial glycocalyx were similar to the effect of 3‐FL. All three tested inulin types with DPs of DP3‐DP10, DP10‐DP60, and DP30‐DP60 increased the average thickness of the albumin layer after 1 day and 5 days stimulation. The short chain DP3‐DP10 inulin that is commonly applied in infant formula already increased the average thickness of HS within 1 day.^45^ Interestingly, DP30‐DP60 inulin selectively increased the average area coverage of HA within 1 day. All these data suggest that inulins have a structure‐dependent effect on the compositions and development of the glycocalyx. Since the enhanced development of the intestinal epithelial glycocalyx layer is associated with expedited colonization with commensal microorganisms,^11^ our results suggest that short chain inulins may be instrumental for this effects and may be further supported with application of long chain inulins as this increases the average area coverage of HA.

The effects were different with pectins than with hMOs and inulins and effects were DM dependent.^1^, ^46^ Only DM7 pectin significantly increased the average area coverage of albumin after 1 day of stimulation. After longer term exposure, that is, 5 days to DM7, DM55, and DM69 pectins a significant increased average thickness of albumin was observed. There was a tendency of pectins to increase the development of HS after 1 day, but this never reached statistical significance. Our observations indicate that the pectins can modulate protein adsorption but has only a minor impact on the carbohydrate components of the glycocalyx layer.

In summary, here we show that 2′‐FL and 3‐FL, and NDCs including inulins and pectins are able to stimulate maturation of the glycocalyx of intestine epithelial cells in a structure‐dependent fashion. Especially hMOs and inulins have such an effect while pectins were less effective. As increased maturation expedites colonization of the infant intestine with microbiota, our data suggest that hMOs and inulins seem both to be effective for glycocalyx development. Our data also contributes to a better understanding of how hMOs and dietary fibers contribute to a healthier gut in infants. It shows that it not only is beneficial for microbiota by serving as carbohydrate source but also directly stimulates maturation of the intestinal anchoring points for bacteria, that is, the glycocalyx.

## Conflict of Interest

The authors declare no conflict of interest.
